# Similarity ratings for basic-level categories from the Nosofsky et al. (2018) database of rock images

**DOI:** 10.3389/fpsyg.2024.1438901

**Published:** 2024-11-08

**Authors:** Bryan White, Eben Daggett, Michael C. Hout

**Affiliations:** ^1^Department of Psychology, New Mexico State University, Las Cruces, NM, United States; ^2^Department of Kinesiology, New Mexico State University, Las Cruces, NM, United States

**Keywords:** similarity, perception, attention, multidimensional scaling, natural categories

Data hosted on the Open Science Framework: https://osf.io/azrmw/.

## Introduction

The concept of similarity is crucial to our exploration and understanding of cognitive processes. For example, by examining how visual attention is differentially distributed to targets and distractors (varying in similarity of appearance), researchers can gain insight into cognitive resource management, template matching, and object recognition. In such experiments, researchers often couple manipulation of similarity among their stimuli with measures of attentional allocation and decision-making (Kahneman and Tversky, 2013). By manipulating the similarity of targets and distractors, or the similarity amongst the set of distractors themselves (Desimone and Duncan, 1995), such research offers insights into the mechanisms of attention, decision-making, and human behavior.

Controlling for stimuli in such experiments can be expensive in labor and time, however, resulting in a tendency to rely upon hyper-simplistic experimental designs; for instance, experimenters wishing to employ a task with highly similar targets and distractors may ask their participants to search for a *7* target among many *T* distractors. Such simplicity is not always preferred (or optimal) however, especially in research necessitating more complexity, such as tasks that use categorically defined targets (Hout et al., 2017; Madrid et al., 2019; Robbins and Hout, 2020), searches set in visually complex environments (Hout et al., 2021, 2023), or dynamic search tasks that are challenging to hyper-simplify (Scarince and Hout, 2018). Questions such as these often require complex stimuli, but when similarity must be manipulated in an experiment, it can be challenging to know how best to do so precisely. One may, for instance, reasonably assume that a *7* is more similar to the letter *T* than it is to the letter *R*; such an assumption is uncontroversial and may suffice if the researcher simply requires pairs of stimuli that are or are not alike. If a researcher is interested in categorical visual search, however—how people look for a class of items (e.g., “search for *any* coffee mug”) rather than a particular exemplar—alphanumeric digits (or other simplistic stimuli) typically do not suffice. Moreover, researchers often require more than a simple dichotomy of similar vs. not similar, and instead need to be able to explore more nuance in their data by obtaining a continuum of similarity among the stimuli (one that can be quantified but is therefore much more subject to individual differences in perception).

When scaling up to pictures of real-world objects, simple assumptions—regarding what objects are more or less similar to one another—are often not sufficient, straightforward, or uncontroversial. Rating feature rich, complex objects for similarity requires a significant empirical investment unto itself, but doing so affords the researcher experimental tools and precision of manipulation that is otherwise impossible to achieve. The suboptimal alternative, of course, is exclusive reliance on hyper-simplistic stimuli for which assumptions about similarity perception are deemed sufficient.

To provide a concrete example: Hout et al. (2014) established a similarity database of ~4,000 visual pictures of real-world items sampled from across 240 distinct categories; the images were sourced from the “Massive Memory Database” (Brady et al., 2008; Konkle et al., 2010; cvcl.mit.edu/MM/stimuli.html). After collecting similarity ratings, they employed multidimensional scaling (c.f., Hout et al., 2013b) to model similarity relationships among the items, providing future researchers with continuous similarity relationships for 16–17 exemplars from each category. Since its initial publication, the similarity ratings from this database have been used to explore various cognitive processes such as attention, template formation, and memory. For instance, Hout and Goldinger (2015) systematically manipulated the similarity between a visual search cue (e.g., “look for this picture or something like it”) and the subsequent item that appeared in the array. Similarity scores from the database (Hout et al., 2014) predicted how efficiently eye movements could be directed to the target, and how quickly the target would be recognized once it was fixated. Guevara Pinto et al. (2020) gave participants a visual search task followed by a surprise alternative forced-choice task in which they were asked to select which of 16 category-matched exemplars was presented to them earlier in the experiment. The authors found that when the correct item was *not* chosen, similarity scores (i.e., the similarity of the incorrectly chosen “foil” relative to the item that had actually been shown previously) predicted the likelihood that an item would be selected. Simply, when subjects made a mistake, they were systematically more likely to choose visually similar foils (and the strength of this relationship was found to be modulated by the number of items that were being looked for).

Because such similarity databases are so useful (but are also relatively rare; see also Horst and Hout, 2016) our goal in this project was to expand upon the natural category stimuli set created by Nosofsky et al. (2018; see also Meagher et al., 2017), by adding to it a set of similarity scores—isolated to each of the basic-level stimuli categories separately, as was done in Hout et al. (2014)—that can be used in cognitive experiments exploring attention, memory, categorization, and more.

### Similarity ratings of the Nosofsky et al. (2018) stimuli

In Nosofsky et al. (2018), 360 rock images were collected for the purpose of studying the learning of complex natural science categories. These stimuli consist of high-resolution images, with each exemplar categorized as belonging to one of the three superordinate categories (based on the processes governing its formation): metamorphic, sedimentary, and igneous. Each superordinate category comprised 10 nested basic-level categories (e.g., marble, granite, obsidian), and each basic-level category contained 12 exemplars (or “tokens”), varying in similarity of appearance, but standardized in form (i.e., all were images of rocks isolated on a blank white background, uniform in overall size).

In one experiment, Nosofsky et al. (2018) selected 30 representative tokens (one from each basic-level category), and had participants rate the similarity of each pair (see also Nosofsky et al., 2017). In a second study, the researchers had participants rate the similarity between each of the 360 instances (from all categories), providing a larger matrix of similarity data, but one that was more sparsely populated (i.e., with fewer similarity ratings per pair of stimuli, given that there are 64,620 unique pairs in a 360 × 360 matrix). What is shared by both studies is the fact that exemplars were compared to one another *across* categories. In the second study, exemplars from *within* the same category were also compared to one another, but only in the context of the entire set of 360 tokens sampled from across 30 categories. Context effects—the tendency of similarity ratings to be determined not just by perceived similarity of a pair but to also be influenced by the participants' understanding of the entire set of stimuli (see Tversky, 1977; Goldstone et al., 1997)—might therefore may have resulted in items from within a single category being perceived as more similar to one another than they would be if that single category was being considered in isolation.

It is inarguable that the data collected by Nosofsky et al. (2018) is useful for studying natural category learning (see Nosofsky et al., 2022, for example, for an application of this dataset to prototype and exemplar models of category formation). What is lacking, however, are complete similarity matrices for each basic-level category collected in a method that is free of potential context effects that might “compress” the perceived similarity of items within a single category. To add to the utility of this dataset, we had participants rate the similarity of all exemplars in a given basic-level category in isolation, allowing them to appreciate the nuance of the perceptual details contained within a category without being affected by the context of the greater set of categories/tokens.

### Similarity rating methods and multidimensional scaling

Frequently, similarity ratings are obtained via the pairwise method of comparison (Thurstone, 2017), whereby participants are tasked with numerically rating item pairs via Likert Scales (Likert, 1932), progressing through every possible pairing in a stimulus set. While the pairwise method has the advantage of task simplicity, a central drawback is that it burdens researchers (and participants) with a costly expense in time and data collection effort. This is especially the case when used for rating large stimulus sets, as the number of needed comparisons grows dramatically with every stimulus added to the set. Because of the length of time required to perform the ratings, researchers must be wary of both rater fatigue and criterion drift, generating concern over data reliability (Johnson et al., 1990).

By contrast, the Spatial Arrangement Method (SpAM) (Goldstone, 1994) presents participants with multiple stimuli at once, tasking the rater with arranging them in space relative to the perceived similarity between each pair. Items that are of high perceived similarity are to be placed proportionally closer together than items considered dissimilar; the data matrix derived from this task simply includes recording Euclidean distances between each pair of items at the end of the arrangement. Whereas the rating of a 12-token set might require 5–10 min for a participant to rate all 66 unique pairs, a single SpAM trial could present all the items at once, and might require only 2–3 min of arrangement to obtain complete ratings. The SpAM task is intuitive, it takes advantage of the natural tendency to think of similarity in a spatial manner, and it is much faster and more efficient than pairwise methods. Indeed, while not right for every experimental scenario (see Verheyen et al., 2016), it has been shown that this speed and efficiency does not come at the cost of data quality for perceptual (Hout et al., 2013a; Hout and Goldinger, 2016) or conceptual (Richie et al., 2020) similarity rating tasks.

Regardless of the similarity rating method used, obtained data are then most frequently subjected to multidimensional scaling (MDS) analysis, a statistical procedure used to model the similarity relationships among items in a set (see Hout et al., 2013b and Hout et al., 2016, for tutorials). The goal of subjecting this “proximity data” (i.e., similarity ratings obtained via pairwise method, SpAM, or some other technique) to MDS is to produce a visually accessible interpretation of the similarity relationships present in the data (which can be useful in hypothesizing as to what features of the stimuli governed participant ratings), and to provide a comprehensive set of “distances” in MDS space that quantify the degree to which any given pair of stimuli are perceived as similar or dissimilar (relative to the other items/pairs in the set).

### The current project

In this project, we collected similarity ratings (via SpAM) for each of the 30 basic-level categories (separately) from the Nosofsky et al. (2018) dataset. Participants rated the similarity of all 12 exemplars in a category on each trial, and data were thereafter subjected to MDS analysis. This data report presents those data and MDS models; full results can be found on our OSF page. It is important to note that this database is intended merely as a supplement to Nosofsky et al. (2018). Specifically, this dataset is intended to focus on the discriminability and similarity of items within each category, which can vary significantly depending on the heterogeneity of the set (e.g., members of the Quartz category are highly similar to one another, whereas those in the granite category vary more in appearance). By collecting similarity ratings within a single category at a time, within-category similarity can be better appreciated, without being “compressed” via comparison to members of other categories (e.g., Quartz items might clump together more than granite in the context of all other categories present in Nosofsky et al., 2018).

The cost of allowing for more nuance in within-category ratings, however, is that we are unable to provide across-category ratings for the stimuli, as have already been provided by Nosofsky et al. (2018). As such, this dataset is likely more useful for studies of visual attention (that necessitate within-category similarity manipulations as in Hout and Goldinger, 2015) than it is for category-learning paradigms that require similarity comparisons across categories. Moreover, our approach does not allow for comparisons across categories (e.g., determination of the relative discriminability of Quartz items relative to granite tokens), and might influence of the impact of “global” features that are common across many categories [e.g., light vs. dark was shown to be a feature of high importance in the Nosofsky et al. (2018) dataset, but for universally dark categories like Obsidian, isolated similarity ratings are unlikely to reveal that featural dimension]. Instead, the value of our dataset is that it provides relatively unperturbed similarity ratings within each category, allowing for subtle differences between tokens to be more impactful when not influenced by the features of other categories.

## Methods

### Ethics

This research was approved by the Institutional Review Board of New Mexico State University (on 7/2/22, IRB Protocol #2206002241), and was considered “exempt” in accordance with Federal regulations, 45 CFR Part 46. Upon arrival, informed consent detailing the aim and scope of the research (as well as the right to refuse or withdraw at any time without consequence) was obtained from all participants in writing. Participants were then instructed how to perform the task and were given an opportunity to ask questions before they began.

### Participants

Thirty-five participants were recruited from the student population at New Mexico State University. Students were compensated with participation credits that could be used as extra credit or as partial fulfillment of course requirements. All participants had normal or corrected-to-normal vision, and all reported normal color vision.

### Design

All participants completed an experiment where they were given 30 SpAM trials (with categories selected in random order) containing 12 stimuli each, taking an average of 50 min to complete. All participants saw and arranged all items within each of the basic-level categories for similarity. In this manner, each item was scaled for similarity against every other item within their respective categories (e.g., all granite exemplars were rated against all other granite exemplars).

### Stimuli

The stimuli used were from the Nosofsky et al. (2018) study. There were a total of 360 stimuli, sampled from across three superordinate categories (metamorphic, sedimentary, igneous), and belonging each to one of 30 basic-level categories (e.g., marble). Each basic level category contained 12 exemplars; all pictures were standardized in format, with a single picture presented across a blank white background, and uniform in size.

### Apparatus

Data were collected on five computers simultaneously, all with identical hardware and software, and supervised by research assistants. The computers used were Dell Inspiron 14 5410 2-in-1 computers with Intel i7 processors running Windows 10 and high-resolution ASUS VP28UQG LCD monitors operating on a 60 GHz refresh rate. The experiment was run on E-Prime v3.0 Professional Software (Schneider et al., 2002).

### Procedure

**Figure 1:**
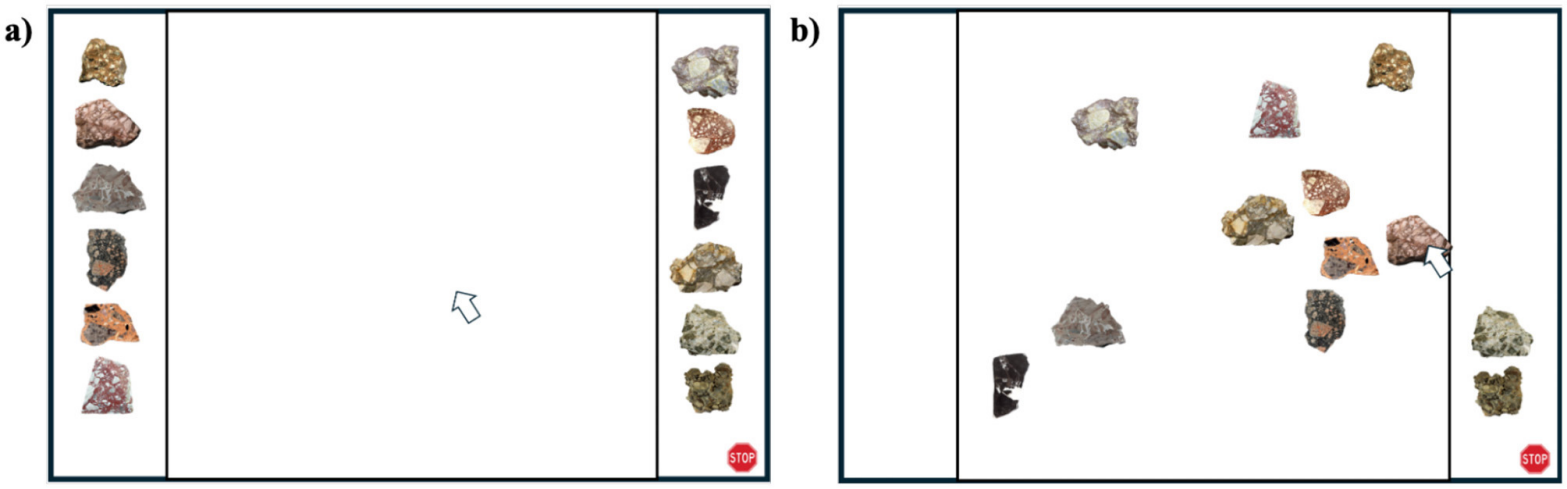
Example progression of a Spatial Arrangement Method (SpAM) trial. **(a)** Trials began with all stimuli from a single category (this one being *breccia*) placed at random on the periphery of the central arena. **(b)** Participants would then drag and drop stimuli into the center, arranging them for similarity with like items placed close together, and dissimilar items placed further apart. When satisfied with their arrangements, they would terminate the trial by clicking on the “Stop” icon at the bottom right of the screen.

In each trial, a new selection of images was shown to the participant on a computer screen (see Figure 1a). The screen was divided vertically by a black border into three distinct areas: left, right, and a central “arena.” The left and right areas on the periphery of the screen were each populated by a row of rock images from top to bottom, placed in random order. Participants were asked to “drag and drop” each item from the periphery into the center arena and arrange them by perceived similarity such that items that were perceived of as similar would be placed proportionately closer together than items perceived as more dissimilar. Samples were not provided to participants because we did not want to bias the participants' perception of the stimuli, and our many prior experiments suggest that users find this interface intuitive and simple. That said, participants were also afforded the opportunity to ask research assistants any questions they had prior to beginning the task in order to ensure that they understood how to utilize the space.

Participants were given as much time on the task as they needed but were not able to advance to the next trial until all items had been removed from the periphery and arranged in the arena (see Figure 1b). Typically, these trials lasted 2–5 min. Upon completion, participants were instructed to click on a Stop Sign icon at the bottom of the screen. To avoid accidental termination of trials, participants were then asked if they were satisfied with their arrangement of the stimuli or if they'd like more time. If they indicated satisfaction with a key press, they were then advanced to the next trial (if not, they were returned to their ongoing trial). The experiment continued in this way through to completion, with participants being allowed to take breaks as needed. Similarity scores were recorded at the end of each trial; we calculated the two-dimensional Euclidean distance between each pair of items at the end of the arrangement and used that information to populate each participants' similarity matrix (for each category). Upon completion, all participants were debriefed on the experimental aims and released.

## Analysis

### Open access materials

All data discussed in the analysis section can be found in the Open Science Framework page (https://osf.io/azrmw/). The page houses a downloadable zip file with four key contents: (1) a spreadsheet containing the database, (2) the R script utilized to conduct the analysis, (3) a folder containing all raw input data for the R script in .csv format, and (4) a folder containing all outputs from the R script in .csv format that were used to construct the database.

### MDS algorithm

For each of the 30 rock categories, we used Kruskal's non-metric multidimensional scaling (MDS) (Kruskal, 1964) to derive a series of MDS coordinate solutions with successively increasing dimensionality. Kruskal's stress formula was used to calculate a stress metric for each dimensional solution within each of the categories. The MDS analysis was conducted in R, utilizing a script (Daggett et al., under review)^1^ that takes the aggregated similarity data collected via SpAM as input and iteratively runs the data through the MDS program at a dimensionality of 1 through *n*, with the experimenter choosing the value of *n* as the maximum dimensionality to be utilized. With each successive iteration of the process, the script produces a coordinate set, a measure of stress, measures of prototypicality (centrality), and a metric called “uniqueness” (detailed below) for each item in the set.

### Dimensionality of the MDS space

A traditional methodology for determining the proper dimensionality for the MDS solution is to analyze the reduction in stress exhibited as the dimensionality of the solution is increased. Such data can be visualized in a *scree plot*. A typical heuristic approach to analyzing the scree plot involves the subjective identification of an “elbow” in the often monotonically decreasing stress value as a function of dimensionality. This elbow represents a point at which an increase in dimensionality represents a diminishing return in the reduction of stress (Kruskal and Wish, 1978). Other approaches for determining dimensionality include the use of Bayesian techniques (Lee, 2001) to more objectively determine an optimal tradeoff between lower, more interpretable dimensionality and stress.

### Classification of item prototypicality (centrality)

**Figure 2:**
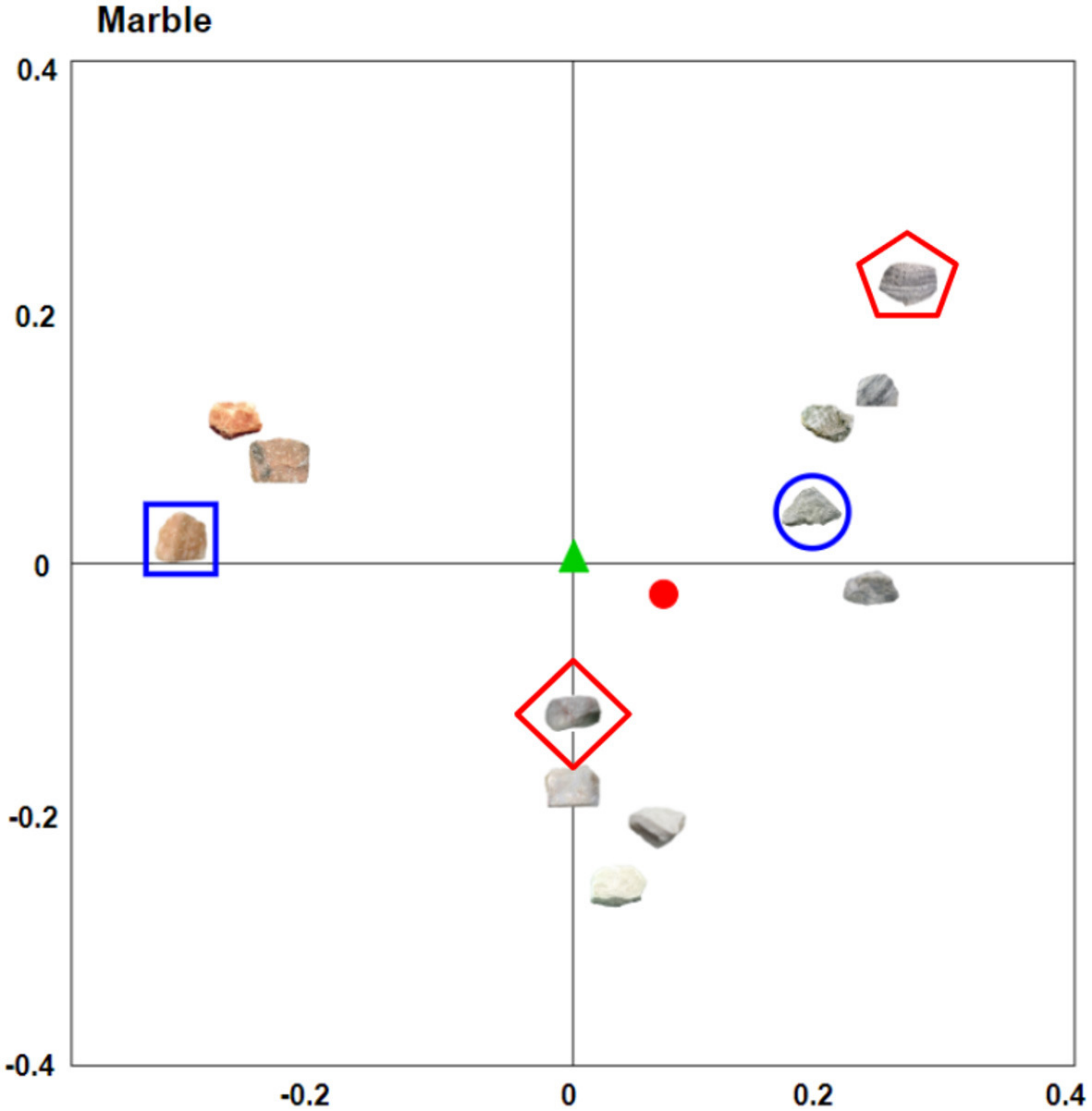
The first two dimensions of the marble category MDS space. Both the geometric center (green triangle) and the centroid (red circle) are depicted for visualization purposes. From the visual, it is possible to see the benefits of utilizing the centroid—or center of mass—over the geometric center, as the centroid accounts for the increased density on the right side of space. The centroid more aptly depicts the orange-tinted marble as being less prototypical for this particular space of marble rocks. The most “unique” rock in the space (blue square)—the rock with the largest average pairwise distance to all other rocks—can be seen in contrast to the least unique rock (blue circle), which has the smallest average pairwise distance. Finally, the most central (red diamond) and most peripheral (red pentagon) rocks are classified based on their distance from the centroid (red circle).

Within the results form, a metric of centrality is provided for each rock in each of the MDS solutions (see Figure 2). Centrality represents a hypothetical measure of “prototypicality” in the context of the set of images being rated, where the more central an object is within the set, the more similar it is to a hypothetical prototypical object. To calculate centrality, we first calculated the center of mass (centroid) of the space of objects for each MDS solution by averaging each of the coordinate dimensions across all objects. We believe this approach to designating the center of the MDS space—as opposed to calculating the geometric center, for instance—is superior in the context of psychological data because it places outlier objects in the peripheral of the space. Consider the generated MDS space of marble rocks (Figure 2), where there is a clear distinction between achromatic (i.e., gray and white rocks) and the orange/red rocks in the stimuli set. The achromatic rocks vastly outnumber the orange/red rocks, and while the geometric center of this space would reside equidistant between all objects, this would not give any consideration to the relatively higher density of stimuli on the right hand (achromatic) side of the space. The centroid of the space, however, does take into account the relative densities within the space. In this example, the centroid would exist farther to the right of the space than the geometric center, resulting in the achromatic rocks being more central than the orange/red rocks.

### Item uniqueness scoring

In addition to centrality scores, the outputs include a metric of “uniqueness”^1^ for each object in each MDS solution (see Figure 2). This scoring represents a novel metric for this stimulus set that offers new avenues by which the images can be leveraged. Uniqueness is a metric that is independent of centrality. Rather than relating each rock to the centroid of the space, uniqueness instead relates each rock to every other rock in the space. To achieve this—for each rock in the space—the mean pairwise distance between a rock and every other rock in the space is calculated. The most unique rock in the space will be the one with the greatest mean pairwise distance between it and any other rock in the space. The least unique rock will be the rock with the smallest average pairwise distance in the total set of pairwise distances in the space.

## Data Availability

The datasets presented in this study can be found in online repositories. The names of the repository/repositories and accession number(s) can be found in the article/supplementary material.

## Footnotes

^1^Daggett, E. W., and Hout, M. C. (under review). A methodology for visualizing high-dimensional psychological spaces without dimensionality reduction. *Behav. Res. Methods*.
